# Three Pivots for Improving Health Care Provider Performance

**DOI:** 10.9745/GHSP-D-21-00625

**Published:** 2022-02-28

**Authors:** Julia Bluestone, Erica Troncoso, Laura Fitzgerald, Lauretta Nagbe, Gladys Tetteh, Augustino Hellar, Edwin Ernest

**Affiliations:** a Jhpiego, Baltimore, MD, USA.; b Jhpiego, Monrovia, Liberia.; c Operation Smile, Dar Es Salaam, Tanzania; formerly of Jhpiego.; d Jhpiego, Dar Es Salaam, Tanzania.

## Abstract

We share recommendations on 3 important pivots away from longstanding approaches to continued professional development and in-service training programs that have demonstrated a measurable benefit across a diversity of health-related applications and projects.

## Key Messages

- The first pivot involves shifting from focusing on using data primarily for reporting purposes. Encouraging team members within facilities to regularly use their own data to track, review, and measure priority quality indicators promotes the quality improvement process and can improve service delivery outcomes.
- The second pivot involves shifting from focusing on repeated, external, quality assurance assessments, often from the district or subnational level, to using facility team-based internal service delivery indicator data reviews to implement quality improvement efforts based on these data.
- These pivots toward health care provider and facility manager ownership of the quality improvement process also lead to the third pivot, which involves shifting from one-time, group-based training toward workplace-based educational interventions. Team involvement in interpreting their facility data can lead to identifying targeted team-based learning needs and the potential need for individual mentoring.

## INTRODUCTION

Many countries invest large portions of their health budgets on standalone, in-service training to enhance skills and performance of health care providers followed by repeated external supportive supervision visits, which often find similar challenges and gaps and result in a low return on investment. An increasing body of evidence points to the limitations of traditional training and supportive supervision to improve performance. A 2016 review of nationally representative surveys in 7 sub-Saharan Africa countries that examined the association of in-service training and supervision with provider quality in antenatal and sick child care found these traditional interventions were associated with only modest improvements.^1^ Similarly, a recent update to a landmark literature review on interventions to improve health care provider performance in low- and middle-income countries (LMICs) found that workplace-based mentorship, clinical practice, and training each had a more positive impact on performance compared to in-service training alone.^2^ Despite this evidence, group-based training and supportive supervision continue to serve as the default interventions to improve health care provider performance, often defined as compliance with care and treatment protocols and ability to deliver quality services that result in improved client health outcomes.^3^ Based on global evidence and Jhpiego’s project experience, we have identified 3 important pivots to improve health care provider performance (Figure). In this commentary, we share these practical pivots for countries and donors to consider for greater return on investments from efforts to improve health care provider performance.

**FIGURE:**
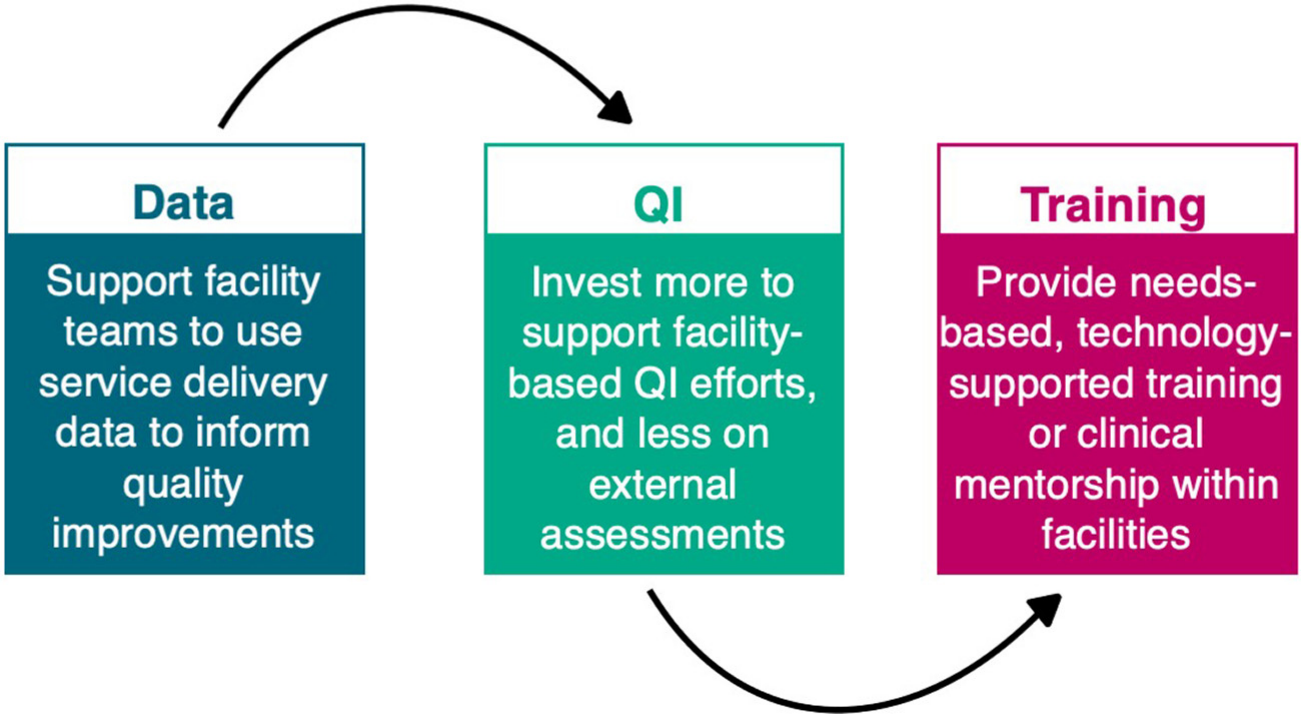
Three Pivots to Support Improved Health Care Provider Performance

Abbreviation: QI, quality improvement.

## PIVOT 1: SUPPORT TEAMS TO USE SERVICE DELIVERY INDICATOR DATA TO DRIVE QUALITY IMPROVEMENT EFFORTS

Subnational management teams invest significant effort collecting data on service delivery indicators to report up the management chain. We recommend investing greater effort to help support teams within facilities collect, review, and use their own priority service delivery indicators to identify gaps and improve performance rather than only report up the management chain. Across several projects, we have seen the power of helping teams within facilities use a few key service delivery indicators to measure quality improvement progress and identify areas where training is needed. The Tanzania Safe Surgery 2020 project, funded by the GE Foundation and ELMA Foundation and implemented from January 2018–December 2020 in the Kagera and Mara regions of Tanzania, used a team-based approach to introduce a package of proven quality improvement and capacity building interventions for surgical teams (interprofessional teams comprised of doctors, nurses, and anesthesia providers) in health care facilities. These approaches included enhancing teamwork, communication, and clinical/surgical skills that focused on team processes to promote a culture of patient safety and introduce infection prevention bundles, safety tools (e.g., the World Health Organization’s Surgical Safety Checklist), regular audit and feedback, process mapping to improve functional organization of services, and regular data review of a few priority service delivery indicators to drive quality improvement efforts. These approaches resulted in significant improvements in health care provider performance and client health outcomes. The Surgical Safety Checklist utilization rate for cesarean deliveries increased from 3.7% (5 of 136) to 95.1% (136 of 143) with *P*<.001. The surgical site infection rate at baseline, before the intervention, was 13.9%. After 18 months of program implementation, this rate declined significantly to 0.7% (a reduction of 94.9%; *P*<.001), showing that the interventions had a positive impact. The same study also found a 38.5% reduction in the perioperative mortality rate related to cesarean deliveries in participating facilities.^4^ Although not statistically significant, these results suggest that the interventions reduced mortality related to surgical complications. The participants noted that the monthly process of regular review and sharing of surgical data was seen as integral to driving improvements in performance.

We have seen the power of helping teams within facilities to use a few key service delivery indicators to measure quality improvement progress and identify areas where training is needed.

Regular data reviews of priority service delivery indicators tracked on facility wall charts and sharing data and learning with other sites were instrumental in identifying the need for training or clinical mentorship, prioritizing problems to address, creating quality improvement plans, and tracking improvements over time. During a team reflection meeting, Dr. Leopold Tibyehabwe, Safe Surgery 2020 team member, commented that beyond the improved performance, the regular data use increased provider confidence and ownership:

*Data is the way we are making the teams active in the sessions. They are engaged in the sessions because they know this is their data. It has really strengthened their confidence.*

As of April 2021, 4 months after the program ended, formerly supported program teams were still sharing their data with each other, indicating a significant change in the manner of data use.

Regular review of a few priority service delivery indicators, tracked and visualized using facility wall charts, was also used to drive action under the Expansion of Malaria Services (EMS) project in Liberia, supported from 2017–2019 by the U.S. Agency for International Development (USAID)-funded Maternal and Child Survival Program. Similar to the Tanzania Safe Surgery 2020 project, facility wall charts were used to help facilities track and regularly review relevant malaria data and key service delivery indicators for quality improvement to target ongoing training and mentorship and to identify other interventions that were needed. At the completion of the project, more than 80% of health workers expressed that the wall charts were user-friendly and more than 90% of the staff were confident in plotting and interpreting 3 doses of intermittent preventive treatment of malaria in pregnancy (IPTp3) data in the wall chart.^5^

## PIVOT 2: INVEST GREATER EFFORT TO SUPPORT CONTINUOUS FACILITY-BASED QUALITY IMPROVEMENT PROCESSES

Across multiple countries, large investments are made in external, supervision visits to assess facility and provider performance, a strategy recognized as not resulting in improved health care provider performance.^1^ There is a need for external supervision, but shifting the balance of funding to invest more in support of facility-based quality improvement efforts and less on repeated external assessment visits may result in better cost efficiencies and greater health care provider performance. Continuous quality improvement, or quality improvement in health care, is defined as^6^:

*a structured organizational process for involving people in planning and executing a continuous flow of improvement to provide quality health care that meets or exceeds expectations.*

Shifting the balance of funding to invest more in support of facility-based quality improvement efforts and less on repeated external assessment visits may result in better cost efficiencies and greater health care provider performance.

In both the Safe Surgery 2020 and the EMS projects, continuous quality improvement approaches were a key intervention supported by as-needed, workplace-based training and/or clinical mentoring, This is supported by literature, which recognizes that this combination of efforts can result in greater effect size.^3^ In-person facilitation of quality improvement processes was provided to health facility managers and facility teams to use service delivery indicator data and data interpretation to identify problems and root causes of those problems and to take actions needed to make quality improvements. This helped health facility staff and managers implement the following quality improvement measures: adequately quantifying sulfadoxine-pyrimethamine with timely requisition at service delivery points to reduce stockouts, and providing pregnant women with health education on the benefits of at least IPTp3 with sulfadoxine-pyrimethamine. Seven months later, DHIS2 data were analyzed to assess uptake of IPTp3. The proportion of IPTp3 uptake increased by 17% from 32% in May 2019 to 46% in November 2019 (PR=17%; 95% confidence interval=5%, 29%; *P*<.0073; z=2.6817).^5^

Under the Safe Surgery 2020 project in Tanzania, continuous quality improvement supported by data review and digital technologies facilitated in-person, virtual, and e-mentoring to support teams within facilities. During the project, in-person Safe Surgery 2020 mentors prioritized data use, supported the development of clinical and nonclinical skills (such as leadership, management, communication), and strengthened the organization of services and implementation of each teams' quality improvement plan. Project ECHO, a partner on the Safe Surgery 2020 project in Tanzania, supported additional weekly virtual mentoring via videoconferencing sessions for 10 sites, with a focus on case review and didactic presentations by clinical mentors. (Project ECHO is a collaborative model of medical education and care management that helps clinicians provide expert-level care to patients wherever they live. It is housed in the University of New Mexico Health Sciences Center.) E-mentoring was provided through WhatsApp groups for mentors and surgical teams that were formed early in the program by region for real-time knowledge exchange, group problem solving, and data and resource sharing. As part of regular data reviews, teams applied continuous quality improvement practices. For example, in response to service delivery indicator data trending in the wrong direction (e.g., lower Safe Surgical Checklist use, increasing infections rates), teams identified the problem, performed root cause analysis, and took corrective actions.

## PIVOT 3: INVEST GREATER EFFORT TO PROVIDE NEEDS-BASED, JUST-IN-TIME WORKPLACE-BASED TRAINING SUPPORTED BY DIGITAL TECHNOLOGY

Invest greater effort to provide needs-based, just-in-time workplace-based or on-site training or clinical mentoring supported by digital technology Despite the lack of evidence to support one-time, group-based trainings, often they are still the default intervention to improve health care provider performance, frequently defined as adherence to clinical treatment protocols or guidelines.^3^ Shifting toward targeted, needs-based training based on health outcomes data and leveraging technology to support more efficient delivery can result in improved health care provider performance and lead to greater cost efficiencies.^2^ Taking into account the evidence on effective learning techniques, including setting, frequency, and media, we found that interactive, case-based learning, with hands-on practice or simulation, delivered in the workplace can improve learning outcomes.^7^ Repeated sessions can be even more effective and computer or mobile-delivered didactic instruction, if appropriately designed for user engagement, can be equally as effective as live instruction.^7^ The EMS project combined the use of the facility wall charts with a 1-day workplace-based training on antenatal care and integrated malaria services and targeted facilities with lower performance, based on service delivery indicators, for site-based clinical mentorship. In both the Liberia EMS and the Safe Surgery 2020 project in Tanzania, formal training was provided on a variety of topics (e.g., clinical updates, data quality and use, quality improvement, and leadership or communication skills) and much of the learning took place in the workplace.

Shifting toward targeted, needs-based training based on health outcomes data and leveraging technology to support more efficient delivery can result in improved health care provider performance and lead to greater cost efficiencies.

The Saving Lives at Birth study in Uganda in 2014 paired Helping Mothers Survive Bleeding after Birth (a workplace-based, low-dose, high-frequency, simulation-based training focused on preventing and managing postpartum hemorrhage) with Helping Babies Breathe (a comparable low-dose, high-frequency module to address birth asphyxia). This was a USAID-funded Saving Lives at Birth Grand Challenges for Development research project. The goal was to build the capacity of providers in all 125 facilities conducting births in 12 districts to deliver these interventions. A 1-day training of the entire team—comprising midwives, nurses, clinical officers, and medical doctors—was conducted at each facility on postpartum hemorrhage. This was repeated 2 months later for birth asphyxia. Facility teams were supported to offer low-dose, high-frequency repeated practice sessions for these critical skills. Across all facilities, there was a 17% reduction in postpartum hemorrhage, 47% reduction in retained placenta, 34% reduction in intrapartum stillbirth, and 62% reduction in early newborn death.^8^

The Safe Surgery 2020 project in Tanzania provides an example of how digital technology expanded the provision of workplace-based clinical mentorship and training and the use of data to target training efforts. Starting in June 2020, in response to lockdowns due to the coronavirus disease (COVID-19) pandemic, the project continued to use the WhatsApp groups to share newly created instructional videos that used anatomical models and conduct videoconference sessions. Teams implemented virtual skills practice and participated in virtual mentorship sessions in their workplace. Each virtual mentoring session started with a review of facility service delivery indicator data and trends related to the selected topic for that session and then addressed challenges and quality improvement interventions the team implemented. The project’s clinical mentors delivered virtual mentorship to networks of 5–6 facilities at each session; during these sessions, each network had data reviews, quality improvement discussions, and a mini-case study or a video demonstration. The instructional video demonstrations were delivered via WhatsApp. The use of video added to the quality and richness of content and allowed learners to return to the content at their own pace and schedule if desired. In addition, providers recorded their skills practice in simulation with anatomical models and shared with clinical mentors for virtual feedback. Use of virtual mentorship allowed the mentor team to have more touch points for review and discussion, while reducing the burden on them to physically travel or leave their workplace to provide support.

We see great potential in the power of leveraging mobile technology to support more efficient, cost-effective workplace-based learning. Delivering efficient, just-in-time learning via mobile devices can reduce in-person instruction time and allow programs to reach greater numbers of providers. Under the Safe Surgery 2020 project, WhatsApp was used to link participating teams within the facility networks for delivering needs-based, user-driven, just-in-time microlearning via YouTube video links and/or job aids. Participating teams also used WhatsApp to obtain peer support, share data, problem-solve difficult clinical cases, and push out links to key instructional content.

Delivering efficient, just-in-time learning via mobile devices can reduce in-person instruction time and allow programs to reach greater numbers of providers.

## CONCLUSION

We recognize that these approaches—using service delivery indicators to drive facility-based continuous quality improvement and providing targeted, workplace-based and technology-supported training and/or clinical mentorship—are not novel. However, we believe the thoughtful combination of these 3 pivots can result in improved health care provider performance and client health outcomes, as well as greater return on investments and more sustainable solutions. As digital technology becomes more widespread in LMICs, it can be used in more, and novel, ways as a valuable tool for new methodologies of training and improving health care provider performance. It is important to note that while digital technology allows for efficiency and effectiveness of these approaches, there is no “one size fits all” technology that results in successful outcomes.

We have seen the promise of combining these effective pivots that focus on data use, quality improvement, and workplace-based training. We recommend that projects consider these impactful pivots when designing interventions that target improving health care provider performance.

## Notes

**Peer Reviewed**
